# Dysregulating PHO Signaling via the CDK Machinery Differentially Impacts Energy Metabolism, Calcineurin Signaling, and Virulence in Cryptococcus neoformans

**DOI:** 10.1128/mbio.03551-22

**Published:** 2023-04-05

**Authors:** Bethany Grace Bowring, Pooja Sethiya, Desmarini Desmarini, Sophie Lev, Lisa Tran Le, Yong-Sun Bahn, Seung-Heon Lee, Akio Toh-e, Nicholas Proschogo, Tom Savage, Julianne Teresa Djordjevic

**Affiliations:** a Centre for Infectious Diseases and Microbiology, The Westmead Institute for Medical Research, Westmead, NSW, Australia; b Sydney Institute for Infectious Diseases, University of Sydney, Sydney, NSW, Australia; c Department of Biotechnology, College of Life Science and Biotechnology, Yonsei University, Seoul, Republic of Korea; d Medical Mycology Research Center, Chiba University, Chiba City, Chiba, Japan; e School of Chemistry, University of Sydney, Sydney, NSW, Australia; f School of Geosciences, University of Sydney, Sydney, NSW, Australia; g Western Sydney Local Health District, Westmead, NSW, Australia; The University of Georgia

**Keywords:** phosphate homeostasis, PHO pathway, Pho80, Pho81, polyphosphate, polyP, calcium, calcineurin, Crz1, fungal virulence, *Cryptococcus neoformans*, fungal pathogenesis, stress response, stress signaling

## Abstract

Fungal pathogens uniquely regulate phosphate homeostasis via the cyclin-dependent kinase (CDK) signaling machinery of the phosphate acquisition (PHO) pathway (Pho85 kinase-Pho80 cyclin-CDK inhibitor Pho81), providing drug-targeting opportunities. Here, we investigate the impact of a PHO pathway activation-defective Cryptococcus neoformans mutant (*pho81*Δ) and a constitutively activated PHO pathway mutant (*pho80*Δ) on fungal virulence. Irrespective of phosphate availability, the PHO pathway was derepressed in *pho80*Δ with all phosphate acquisition pathways upregulated and much of the excess phosphate stored as polyphosphate (polyP). Elevated phosphate in *pho80*Δ coincided with elevated metal ions, metal stress sensitivity, and a muted calcineurin response, all of which were ameliorated by phosphate depletion. In contrast, metal ion homeostasis was largely unaffected in the *pho81*Δ mutant, and P_i_, polyP, ATP, and energy metabolism were reduced, even under phosphate-replete conditions. A similar decline in polyP and ATP suggests that polyP supplies phosphate for energy production even when phosphate is available. Using calcineurin reporter strains in the wild-type, *pho80*Δ, and *pho81*Δ background, we also demonstrate that phosphate deprivation stimulates calcineurin activation, most likely by increasing the bioavailability of calcium. Finally, we show that blocking, as opposed to permanently activating, the PHO pathway reduced fungal virulence in mouse infection models to a greater extent and that this is most likely attributable to depleted phosphate stores and ATP, and compromised cellular bioenergetics, irrespective of phosphate availability.

## INTRODUCTION

The AIDS-related fungal pathogen Cryptococcus neoformans causes fatal meningitis (1–3) and has recently been listed by the WHO as a priority fungal pathogen (https://www.who.int/publications/i/item/9789240060241). Treatment involves initiation therapy using amphotericin B, which is often administered in combination with flucytosine, followed by azole maintenance therapy (3). However, amphotericin B is toxic and C. neoformans is becoming resistant to the fungistatic azole drug classes (4, 5). Furthermore, C. neoformans does not succumb to the killing effect of the newer echinocandin class of antifungals, which targets cell wall synthesis (6).

Infection by C. neoformans starts in the lungs following the inhalation of infectious propagules before spreading to the brain via the blood. To survive within the host, fungi must acquire the essential macronutrient phosphate (P_i_) from the host environment, which is required for membrane and nucleic acid synthesis, energy generation, regulation of protein activity through posttranslational modification (7), and synthesis of inositol polyphosphates (8–10), which signal P_i_ availability (11, 12). In contrast to multicellular organisms, fungal cells experience rapid changes in P_i_ availability and have evolved a phosphate acquisition (PHO) signaling pathway to maintain P_i_ homeostasis (7, 13). The absence of a PHO pathway in mammalian cells highlights PHO signaling components as potential antifungal drug targets and the need to better understand PHO pathway regulation and its impact on virulence.

Model yeasts, including Saccharomyces cerevisiae, have provided a platform to identify and characterize PHO pathway components in fungal pathogens (7, 13–15). The PHO pathway activation mechanism in S. cerevisiae and C. neoformans is mostly conserved: a cyclin-dependent kinase (CDK) complex comprised of the kinase Pho85, the cyclin Pho80, and the CDK inhibitor Pho81 (16–20) responds to changes in intracellular P_i_ levels. When P_i_ is abundant, Pho85 is active and phosphorylates the transcription factor Pho4 facilitating the export of Pho4 out of the nucleus. When P_i_ is scarce, Pho81 inhibits Pho85 and dephosphorylated Pho4 remains predominantly localized in the nucleus. This leads to the induction of genes involved in the mobilization of P_i_ from organic sources and the uptake of free P_i_ (16, 17, 21, 22). Another Pho4-responsive gene is *VTC4*, which encodes a component of the vacuolar transport chaperone (VTC) complex involved in the polymerization and storage of excess P_i_ into polyphosphate (polyP) (21).

Despite the similarities between fungal PHO signaling machinery, some differences have been reported. For example, there is a lack of sequence similarity between cryptococcal Pho4 and other fungal Pho4 proteins, which coincides with the absence of the Pho4 transcriptional coregulator (Pho2) and expansion of PHO gene targets in C. neoformans to include transporters of nutrients other than P_i_ to potentially promote adaptation to a host environment (18). Additionally, the inositol pyrophosphate 5-PP-IP_5_, rather than 1-PP-IP_5_, is the predominant inositol pyrophosphate isoform in C. neoformans that is involved in PHO pathway regulation (20). It has been proposed that the level of cytosolic P_i_ is translated into changes in the levels of inositol pyrophosphates, which bind to SPX (Syg1/Pho81/Xpr1) domains (12, 23, 24). This includes an SPX domain within the CDK inhibitor Pho81, where 5-PP-IP_5_ interaction stabilizes Pho81 and its association with the other components of the CDK complex (20).

Studies linking the PHO pathway to virulence have been performed in C. albicans and C. neoformans. Deleting the gene encoding the transcription factor Pho4 in both C. neoformans and C. albicans blocked PHO pathway activation in response to P_i_ deficiency and led to attenuated virulence (16, 19, 20, 25, 26). However, Pho4 had a more prominent role in stress protection and virulence in C. albicans. Loss of Pho4 in C. albicans affected metal cation toxicity, accumulation and bioavailability, and oxidative stress sensitivity (25, 26) while in C. neoformans, *pho4*Δ was only sensitive to high metal ion concentrations and oxidative and nitrosative stress during P_i_ deprivation, and the effect was reversed by P_i_ replenishment (16). In C. neoformans, the loss of all three P_i_ transporter proteins (21) and the secreted acid phosphatase Aph1 (17), all of which are Pho4-responsive genes, also led to reduced virulence. We recently demonstrated that loss of the CDK inhibitor Pho81 had a greater impact on virulence attenuation than the loss of Pho4, as *pho81*Δ was avirulent in a mouse inhalation model of cryptococcosis (20). Similar to *pho4*Δ (16), *pho81*Δ could not activate its PHO pathway, and consequently, induction of all PHO genes was repressed (20). However, other *pho81*Δ phenotypes responsible for the observed loss of virulence were not investigated.

Phosphate is also important for cell signaling. Components of numerous fungal signaling pathways involved in nutrient sensing and stress adaptation are either kinases or phosphatases and/or regulated by phosphorylation and dephosphorylation (27, 28). Calcineurin is a Ca^2+^/calmodulin-dependent protein phosphatase that upregulates the expression of stress response genes including those encoding glutathione *S*-transferases (GST) and catalases to combat heat, endoplasmic reticulum, oxidative, and cell wall stress and promote fungal growth and virulence (29–33). Calcineurin can be inhibited by the immunosuppressant FK506 (tacrolimus), which has antifungal properties. A regulatory connection between the PHO and calcineurin pathways was observed in C. neoformans (21). Given that FK506 is a commonly administered immunosuppressant and invasive fungal infections occur predominantly in immunosuppressed individuals, understanding how the calcineurin pathway intersects with the PHO pathway could inform new antifungal drug development directed toward disrupting fungal P_i_ homeostasis.

As components of the PHO signaling machinery are unique to fungi and could be exploited for antifungal drug development, we characterized the cellular phenotypes of *pho80*Δ, which has a constitutively activated PHO pathway, and *pho81*Δ, which cannot activate its PHO pathway in response to P_i_ deprivation. We also explored the connection between PHO and calcineurin signaling. Elevated P_i_ in *pho80*Δ coincided with a defect in metal ion homeostasis, a muted calcineurin activation response, and attenuated virulence in mouse infection models. Interestingly, intracellular P_i_ (especially polyP) and ATP were depleted in *pho81*Δ, and cellular bioenergetics was compromised, even when P_i_ was present in the growth medium, potentially accounting for why *pho81*Δ is avirulent in a mouse infection model.

## RESULTS

### Loss of Pho80 leads to constitutive activation of the PHO pathway and storage of excess phosphate as polyP.

**FIG 1:**
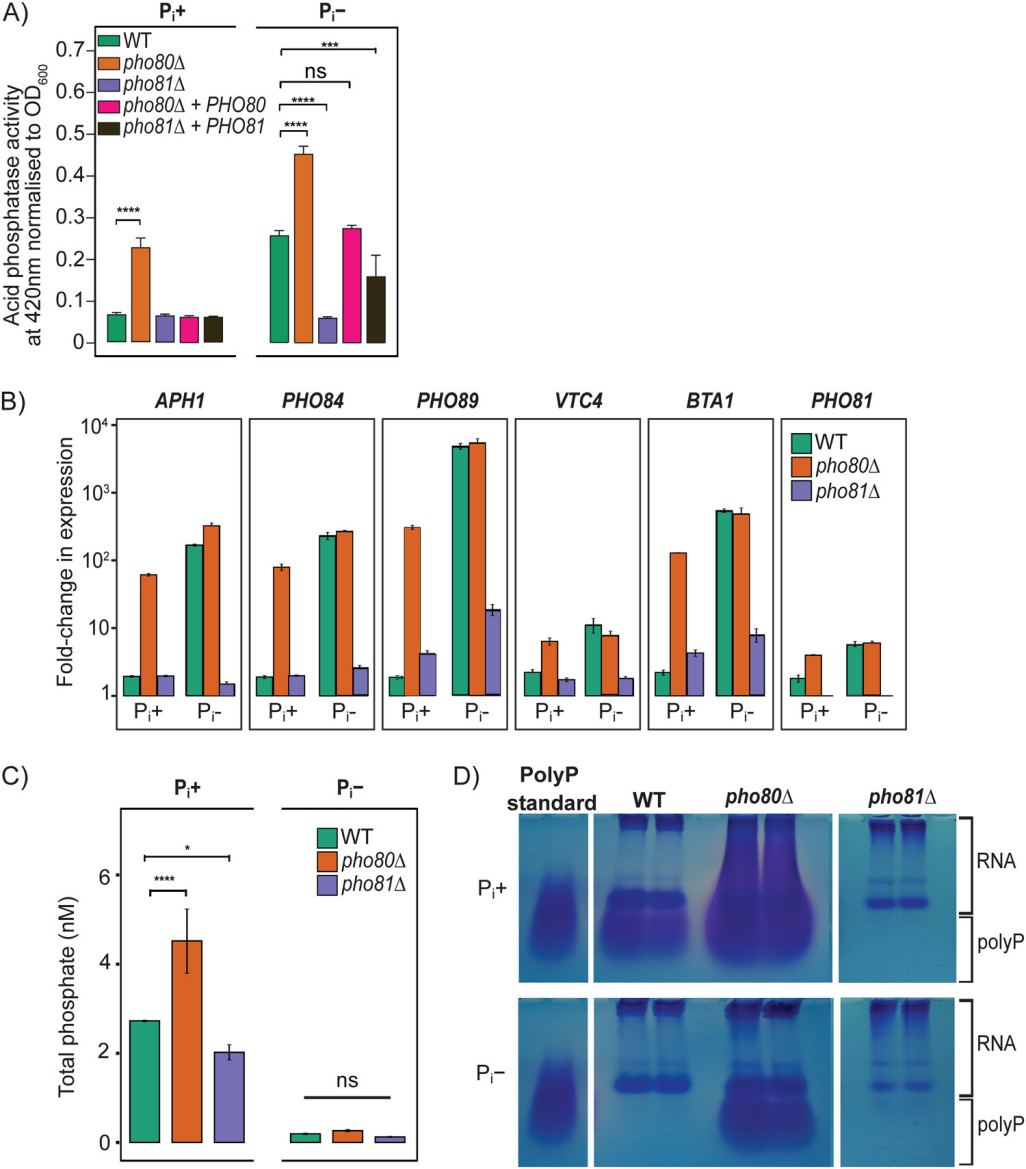
The PHO pathway is constitutively activated in *pho80*Δ leading to phosphate and polyP accumulation. (A) Extracellular acid phosphatase activity derived from Aph1 is constitutively upregulated in *pho80*Δ, in both MM with P_i_ (P_i_^+^) and MM without P_i_ (P_i_^−^). Results are expressed as mean acid phosphatase activity (OD_420_) normalized to cell density (OD_600_) ± standard deviation (*n* = 3 biological replicates). Significance was determined by two-way ANOVA with Tukey’s test for multiple comparisons (ns, not significant; ***, *P* < 0.001; ****, *P* < 0.0001). (B) PHO genes are constitutively upregulated in *pho80*Δ irrespective of P_i_ status but not in *pho81*Δ in the absence of P_i_ over 3 h in MM. Mean ± standard deviation of fold change as calculated by the FC = 2^−ΔΔCt^ method using *ACT1* as the reference gene and normalized to expression of WT in MM with P_i_ (*n* = 3 technical replicates). (C) Total P_i_ levels are increased in *pho80*Δ and decreased in *pho81*Δ in P_i_^+^ media. Mean ± standard deviation of total P_i_ after overnight incubation in MM with and without P_i_ (*n* = 3 technical replicates). Significance was determined by one-way ANOVA and Tukey’s test for multiple comparisons (ns, not significant; *, *P* < 0.05; ****, *P* < 0.0001). (D) PolyP levels are increased in *pho80*Δ and decreased in *pho81*Δ in MM with and without P_i_. PolyP standard was run on the same gel as WT and *pho80*Δ. Panel D polyP standard is duplicated to show the location of polyP in each row.

We created a *PHO80* deletion mutant (*pho80Δ*) and its complemented strain (*pho80Δ + PHO80*) and used the acid phosphatase (Aph1) reporter assay to measure PHO pathway activation (Fig. 1A). In C. neoformans, *APH1* is a highly induced PHO gene during P_i_ deprivation and is located in vacuoles and the external milieu following its Sec14 and endosome-dependent transport through the secretory pathway (17, 34). Secreted Aph1 hydrolyses P_i_ from organic sources, and this P_i_ is taken up by P_i_ transporters to replenish intracellular P_i_ stores (17). In contrast to *pho81Δ*, where no elevation in Aph1 activity was observed in P_i_^−^ medium compared to P_i_^+^ medium (*P* > 0.999), wild-type (WT) cells grown in P_i_^−^ medium had a 3.8-fold increase in Aph1 activity compared to WT cells grown in P_i_^+^ medium (Fig. 1A; *P* < 0.0001). Compared to WT, the PHO pathway in *pho80Δ* was constitutively activated in P_i_^+^ medium with a 3.4-fold increase in Aph1 activity relative to WT (*P* < 0.0001) and hyperactivated in P_i_^−^ medium with a 1.8-fold increase in Aph1 activity relative to the WT in P_i_^−^ medium (*P* < 0.0001). The reintroduction of *PHO80* into *pho80*Δ abolished the constitutive PHO pathway activation response observed in P_i_^+^ medium and reduced the PHO pathway hyperactivation response observed in P_i_^−^ medium (Fig. 1A). The reintroduction of *PHO81* into *pho81*Δ resulted in a partial restoration of WT levels of PHO pathway activation in response to P_i_ deprivation (Fig. 1A).

A more comprehensive assessment of PHO pathway activation in *pho80*Δ and *pho81*Δ was performed using quantitative reverse transcription-PCR (qRT-PCR) to quantify the expression of *APH1* and other PHO genes (16, 17, 21, 22) (Fig. 1B). PHO gene expression was significantly elevated in WT in P_i_^−^ medium compared to WT in P_i_^+^ medium: for WT in P_i_^−^ medium, expression was ~170-fold higher for *APH1*; ~250-fold higher for the high-affinity P_i_ transporter encoding gene *PHO84*; ~450-fold higher for the lipid remodeling enzyme encoding gene *BTA1* (17, 21, 22); ~5,500-fold higher for *PHO89*, which encodes another high-affinity P_i_ transporter (21); ~6-fold higher for the CDK inhibitor *PHO81* (20); and ~8-fold higher for *VTC4* (21), which encodes a component of the vacuolar transport chaperone complex involved in polyphosphate (polyP) polymerization and storage. All PHO genes were expressed constitutively in *pho80Δ* in P_i_^+^ medium and are similar to WT levels during normal PHO pathway activation in P_i_^−^ medium. Basal expression of Pho4-responsive genes was mostly WT like in *pho81Δ* in the presence of P_i_, with the exception of *PHO89* and *BTA1*, and there was little induction of most Pho4-responsive genes during P_i_ deprivation, again with the exception of *PHO89* and *BTA1*. This is consistent with a compromised PHO pathway as observed for *pho4*Δ (16).

Consistent with the constitutive activation of the PHO pathway in *pho80*Δ in P_i_^+^ medium, the level of intracellular free P_i_ was higher in *pho80*Δ than in WT. Interestingly, *pho81Δ* had a lower level of P_i_ compared to WT under P_i_ growth conditions where PHO pathway activation is normally repressed (Fig. 1C). As expected, intracellular P_i_ levels were drastically reduced for all strains in P_i_^−^ media compared to P_i_^+^ media, with no significant differences between strains in P_i_^−^ media (Fig. 1C). In fungi, excess P_i_ is stored as polyP (24, 25). We therefore coextracted polyP with nucleic acids from cell lysates and semiquantified polyP by gel electrophoresis (Fig. 1D). Significant quantities of polyP were observed in WT following growth in P_i_^+^ medium, and the level of polyP was depleted in P_i_^−^ medium. In contrast to WT, *pho80Δ* accumulated polyP in P_i_^−^ medium and even more so in P_i_^+^ medium. This is consistent with an elevated level of P_i_ due to the constitutive expression of PHO genes, including *VTC4*. Vtc4 is a component of the vacuolar transport chaperone (VTC) complex, which is responsible for producing polyP from cytosolic ATP and translocating the nascent polyP chain into the lumen of the vacuole (35). In contrast, *pho81*Δ had almost no stored polyP, even when P_i_ was available (Fig. 1D).

### Impact of altering P_i_ homeostasis on cellular bioenergetics.

**FIG 2:**
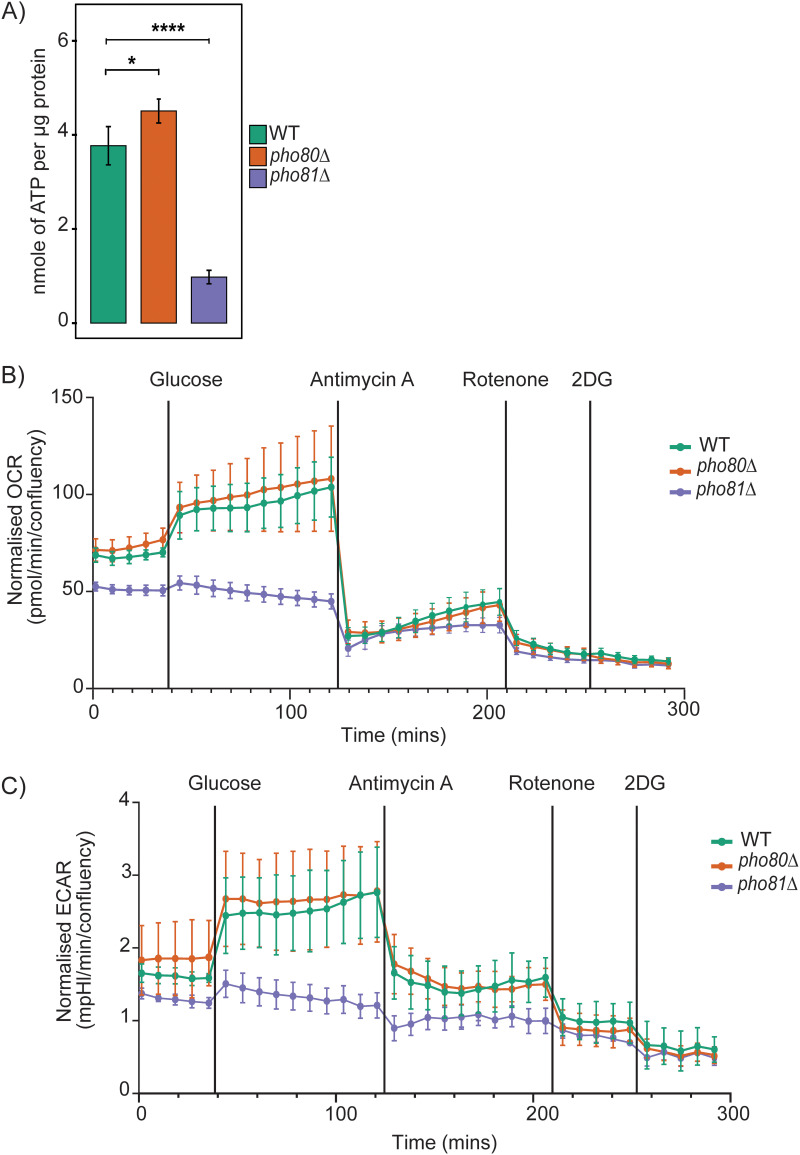
Effect of modulating PHO pathway activation on ATP levels, mitochondrial function, and glycolysis. (A) ATP levels are reduced in *pho81*Δ and increased in *pho80*Δ following a 3-h incubation in MM with P_i_, depicted as mean ± standard deviation of ATP normalized to protein amount (*n* = 3 biological replicates). Significance was determined by one-way ANOVA with Dunnett’s T3 test for multiple comparisons (*, *P* < 0.05; ****, *P* < 0.0001). (B and C) The metabolism of cells seeded onto poly-l-lysine coated wells was measured using Seahorse XFe24 Analyzer instrument (Agilent). Basal metabolic activity was measured for up to 40 min. Thereafter, the changes in metabolic activity were measured for up to 80 min after each compound addition. The compounds added were 20 mM glucose, 50 μM antimycin A, 50 μM rotenone, and 100 mM 2-deoxy-d-glucose (2DG). (B) Oxygen consumption rate (OCR) normalized to cell confluence was reduced in *pho81*Δ compared with WT. Results show the mean ± SEM of OCR of at least 3 wells. (C) Extracellular acidification rate (ECAR) normalized to cell confluence was reduced in *pho81*Δ compared with WT. Results show the mean ± SEM of ECAR of at least 3 wells.

The availability of free phosphate is essential for the generation of energy as phosphate is required to be incorporated into ATP. We therefore measured ATP levels and cellular metabolism in the PHO signaling mutants (Fig. 2). ATP levels were mildly higher in *pho80*Δ compared to the WT but were reduced in *pho81Δ* (Fig. 2A). While some ATP is generated through glycolysis in the cytosol, most ATP is generated through oxidative phosphorylation in mitochondria. We therefore assessed the extracellular acidification rate (ECAR) and the oxygen consumption rate (OCR), which are measures of glycolysis and mitochondrial function, respectively, in both *pho80*Δ and *pho81Δ*, before and after glucose addition using the Seahorse analyzer. Glycolysis and oxidative phosphorylation are tightly coupled in C. neoformans due to its status as a nonfermenting obligate aerobe (36). Hence glycolysis and oxidative phosphorylation cannot be impacted independently of the other. C. neoformans therefore has a limited capacity to restore NAD^+^ via fermentation and, compared to mammalian cells, responds differently to metabolic inhibitors. We therefore had to adapt the standard mitochondrial stress test and glycolysis rate test developed for mammalian cells to measure metabolic flux in C. neoformans as described in references 36, 37. This modified protocol involves equilibrating fungal cells in glucose-free medium to obtain a stable basal metabolic rate; injecting glucose to stimulate metabolic activity; sequentially injecting antimycin A and rotenone to block cellular respiration at complex III and complex I, respectively; and injecting 2-deoxy-d-glucose to block the early steps in glycolysis.

Using our adapted Seahorse protocol to measure OCR, we showed that WT and *pho80*Δ had similar basal OCR and a similar OCR postglucose addition (Fig. 2B). In contrast, *pho81*Δ had a significantly lower OCR (basal and postglucose addition) compared to WT and *pho80*Δ (Fig. 2B). Injection of antimycin A reduced OCR in all strains to levels that were well below basal OCR. A mild increase in OCR was observed in all strains over time postinjection of antimycin A, which was ameliorated following injection of rotenone. Injection of 2-deoxyglucose caused no further decline in OCR in all strains.

In C. neoformans, ECAR is predominantly a measure of glycolysis and glycolysis side-branching pathways, which lead to the production of acetate and protons that are extruded via plasma membrane H^+^-ATPase (38). Some media acidification can also arise from TCA cycle activity due to hydration of CO_2_ to form carbonic acid. Using our adapted Seahorse protocol to measure ECAR, we demonstrate that WT and *pho80*Δ had a similar ECAR (basal and postglucose addition), while the ECAR for *pho81*Δ was significantly lower (basal and postglucose addition) (Fig. 2C), matching its lower OCR profile (Fig. 2B). Addition of antimycin A and rotenone caused steeper reductions in the WT and *pho80*Δ ECAR than in *pho81*Δ ECAR, consistent with the already suppressed mitochondrial function in *pho81*Δ as indicated in Fig. 2B. Injection of 2-deoxy-d-glucose reduced ECAR to the same level in all strains. Overall, the results in Fig. 2 indicate that energy production is significantly reduced in *pho81*Δ, coinciding with reduced levels of P_i_, polyP, and ATP, while WT and *pho80*Δ had similar metabolic profiles (Fig. 2B and C).

### *pho80*Δ, but not *pho81*Δ, accumulates metal ions and is metal ion sensitive.

**FIG 3:**
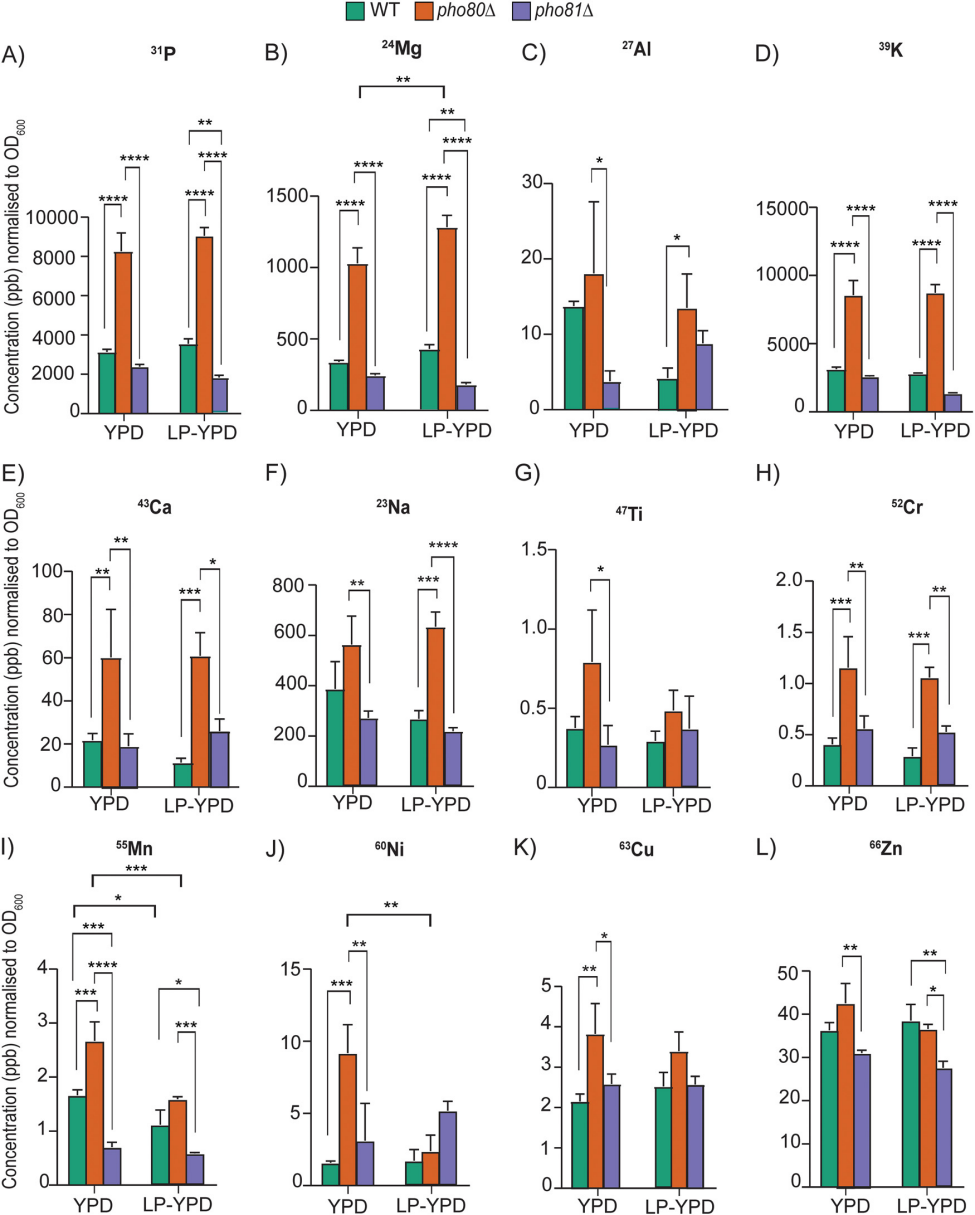
*pho80*Δ cells accumulate metal ions as measured by ICP-MS. Strains were grown overnight in YPD or LP-YPD at 37°C and treated with nitric acid. Elemental analysis by ICP-MS was performed on the lysates in biological triplicate. The results depict the mean ± standard deviation of metal ion concentration in parts per billion (ppb) for each strain normalized to cell density (OD_600_). Results were compared to WT using a one-way ANOVA and Tukey’s test for multiple comparisons (*, *P* < 0.05; **, *P* < 0.01; ***, *P* < 0.001; ****, *P* < 0.0001). (A to L) The elements analyzed were phosphorus (^31^P) (A), magnesium (^24^Mg) (B), aluminum (^27^Al) (C), potassium (^39^K) (D), calcium (^43^Ca) (E), sodium (^23^Na) (F), titanium (^47^Ti) (G), chromium (^52^Cr) (H), manganese (^55^Mn) (I), nickel (^60^Ni) (J), copper (^63^Cu) (K), and zinc (^66^Zn) (L).

Phosphate, and in particular polyP, is involved in the sequestration and storage of cations (21, 39–41), and elevated phosphate due to constitutive activation of the PHO pathway in the *pho80*Δ mutant of S. cerevisiae coincided with a wide range of metal ion homeostasis defects characterized by elevated metal ion levels and/or susceptibility to those metal ions (40). We therefore measured metal ion levels in WT and PHO mutant cell lysates using inductively coupled plasma mass spectrometry (ICP-MS). Given that S. cerevisiae *pho80*Δ hyperaccumulates P_i_ up to 10-fold in rich medium (yeast extract-peptone-dextrose [YPD]) compared to only 50% in minimal medium (40), we grew the C. neoformans PHO mutants in YPD. We also tested metal accumulation in low-phosphate (LP)-YPD, where 65% of the P_i_ content was depleted. This contrasts with the complete absence of P_i_ in minimal media (Fig. 1). In agreement with the biochemical assay for P_i_ and polyP (Fig. 1C), *pho80*Δ accumulated total phosphorous (Fig. 3A). In comparison to WT, *pho80*Δ also accumulated several metal ions including magnesium (Fig. 3B), aluminum (Fig. 3C), potassium (Fig. 3D), calcium (Fig. 3E), sodium (Fig. 3F), chromium (Fig. 3H), manganese (Fig. 3I), nickel (Fig. 3J), and copper (Fig. 3K). *pho80*Δ accumulated more titanium (Fig. 3G) and zinc (Fig. 3L) than *pho81*Δ but not WT. In contrast, *pho81*Δ, which is depleted in P_i_ and polyP, did not accumulate metal ions (Fig. 3).

When *pho80*Δ and *pho81*Δ cells were grown in YPD medium depleted of P_i_ (LP-YPD), total cellular phosphorous was unchanged (Fig. 3A). However, this is most likely due to significant amounts of P_i_ still being present in LP-YPD. In contrast, cellular P_i_ levels were significantly reduced when grown in minimal media without P_i_ (Fig. 1C). Following growth in LP-YPD, manganese (Fig. 3I) and nickel (Fig. 3J) levels declined in *pho80*Δ relative to YPD-grown *pho80*Δ, consistent with a partial rescue by P_i_ deprivation in LP-YPD. However, levels of magnesium (Fig. 3B) were increased in LP-YPD grown *pho80*Δ compared to LP-YPD grown WT. This inconsistency could be due to incomplete depletion of P_i_ from the medium.

**FIG 4:**
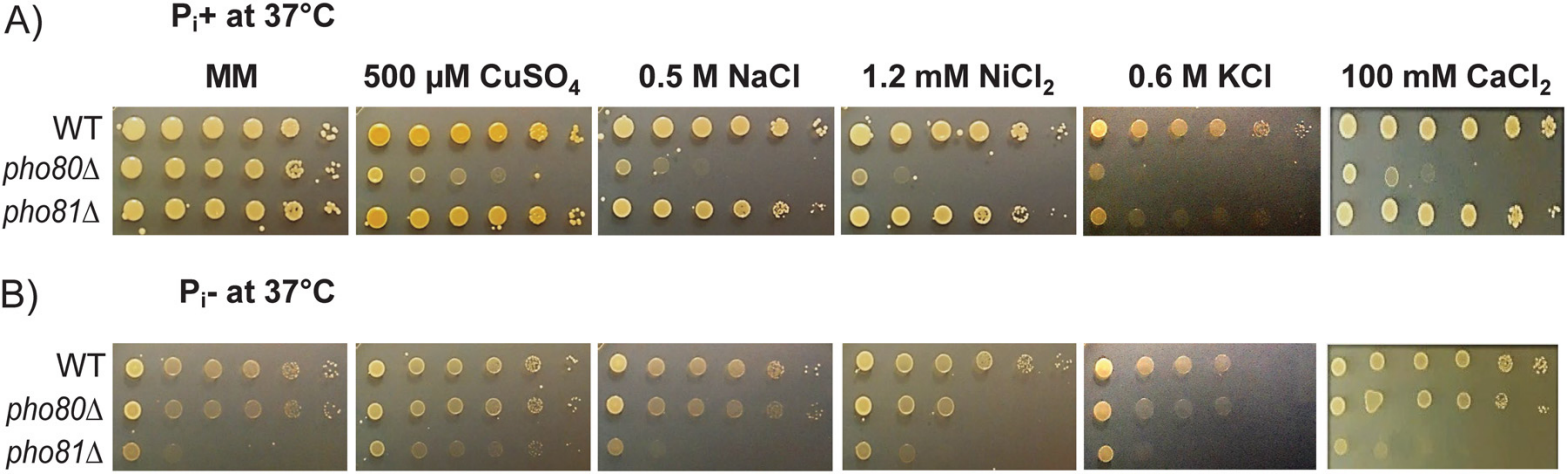
*pho80*Δ is sensitive to high concentrations of metal ions, including calcium, and is rescued by P_i_ deprivation. Strains were grown overnight in YPD and serial 10-fold dilutions were prepared. Three-microliter aliquots of each dilution (containing 10^6^ to 10^1^ cells) were spotted onto MM agar plates containing 500 μM CuSO_4_, 0.5 M NaCl, 1.2 mM NiCl_2_, 0.6 M KCl, and 100 mM CaCl_2_ and grown for 3 days at 37°C in the presence (A) of P_i_ (P_i_^+^) or in the absence (B) of P_i_ (P_i_^−^). Control MM plates are duplicated in Fig. 5C and Fig. 6A as drop tests were performed simultaneously.

We also investigated the sensitivity of *pho80*Δ cells to high concentrations of metal ions and showed that, when P_i_ was present, *pho80*Δ was sensitive to the metals it had accumulated. This included copper, sodium, nickel, potassium, and calcium (Fig. 4A). In contrast, *pho81*Δ was not sensitive to these metals. P_i_ depletion rescued copper, sodium, and calcium ion sensitivity in *pho80*Δ and partially rescued nickel and potassium ion sensitivity (Fig. 4B). As expected, *pho81*Δ growth was suppressed in the absence of P_i_ as *pho81*Δ already has low levels of P_i_ and is unable to activate the PHO pathway to replenish intracellular P_i_ stores (20). In summary, elevated levels of P_i_ and polyP coincide with accumulation, and sensitivity to, a broad range of metal ions.

### Perturbing P_i_ homeostasis impacts calcineurin activation.

A regulatory association between the PHO and calcineurin pathways has previously been reported in S. cerevisiae and C. neoformans (21, 42, 43). Calcineurin is a Ca^2+^-calmodulin-dependent phosphatase, which in C. neoformans is activated predominantly by heat stress, but also by exogenous Ca^2+^ and cell wall stress (30). This leads to dephosphorylation of the transcription factor Crz1 and its translocation to the nucleus (30, 44) to induce the expression of genes involved in cell wall integrity and response to oxidative stress (30–32). To further explore the connection between the PHO and calcineurin pathways, we investigated whether altering cellular P_i_ affects the calcineurin activation response. We created *pho80*Δ Crz1-GFP and *pho81*Δ Crz1-GFP reporter strains from a previously characterized WT Crz1-GFP strain (30). The calcineurin activation response was then assessed in all three strains following growth in the presence and absence of P_i_.

**FIG 5:**
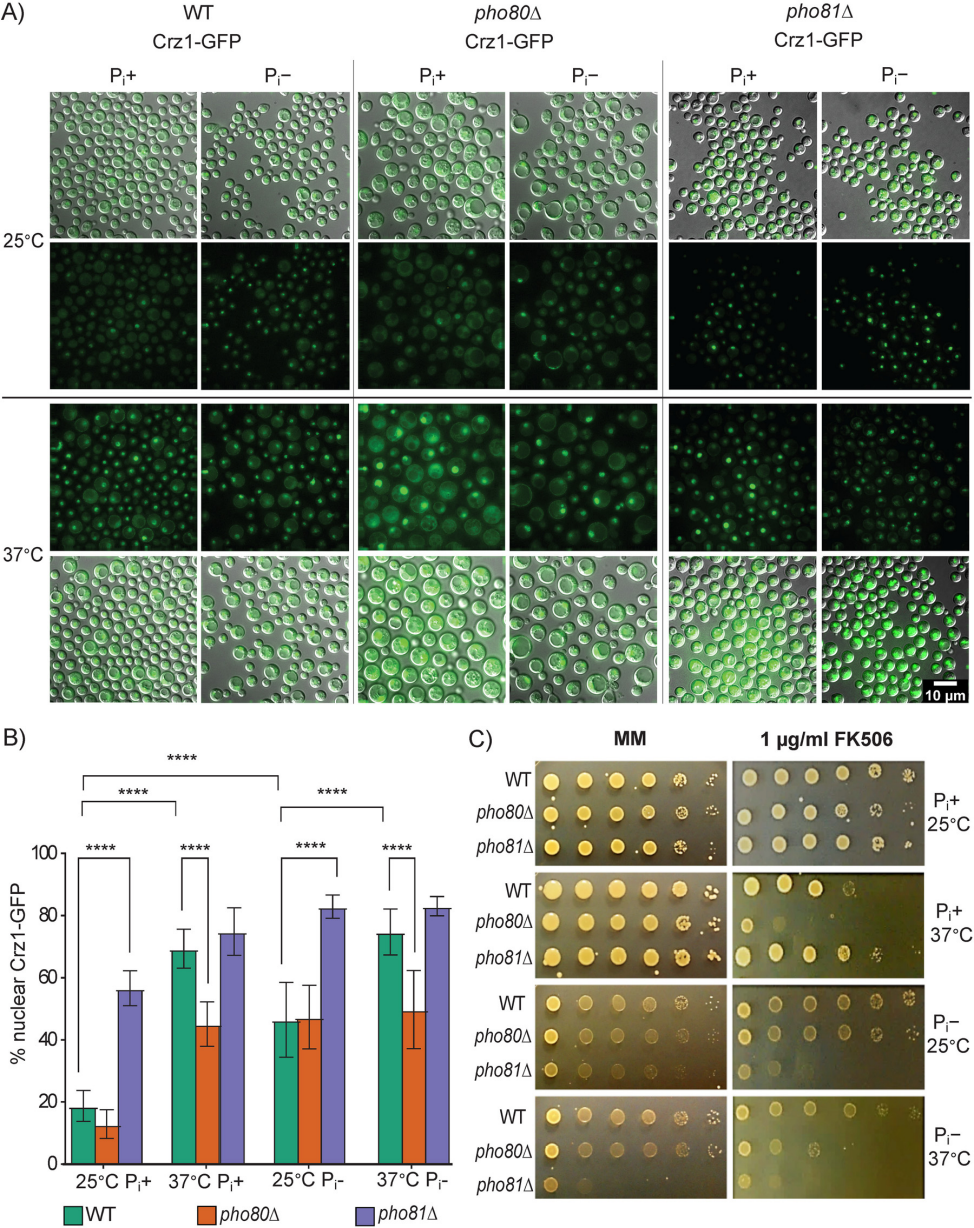
Calcineurin activation is suppressed in *pho80*Δ and triggered by P_i_ deprivation. (A) WT, *pho80*Δ, and *pho81*Δ expressing Crz1-GFP as a reporter for calcineurin activation, were incubated for 4 h at 25°C or 37°C in MM with and without P_i_, as indicated, and Crz1-GFP was visualized using a Delta Vision fluorescence microscope. (B) The percentage of nuclear-localized Crz1-GFP was higher in WT and *pho80*Δ cells at 37°C compared to at 25°C in media containing P_i_ (P_i_^+^). However, the amount of nuclear-localized Crz1-GFP in *pho80*Δ at 37°C was not as high as in WT. In contrast, *pho81*Δ, which is low in P_i_ and depleted of polyP, contained a higher proportion of nuclear-localized Crz1-GFP irrespective of P_i_ availability and temperature. When P_i_ was depleted (P_i_^−^), all strains contained a higher proportion of nuclear-localized Crz1-GFP irrespective of temperature. Results are expressed as mean percentage of cells with nuclear Crz1-GFP ± the standard deviation (*n* > 5 fields of view with >250 cells per strain). Differences in Crz1-GFP nuclear localization among strains were determined by two-way ANOVA with Tukey’s test for multiple comparisons (****, *P* < 0.0001). (C) FK506 susceptibility was assessed for 3 days at 25°C or 37°C in the presence and absence of P_i_ on MM using a spot dilution assay as described in Fig. 4. Control MM plates are duplicated in Fig. 4 and Fig. 6A as drop tests were performed simultaneously.

As previously reported (30), the proportion of WT cells containing nuclear-localized Crz1-GFP at 25°C was low (around 19%) in the presence of P_i_ (Fig. 5A and B). The proportion of *pho80*Δ cells containing nuclear-localized Crz1-GFP at 25°C in the presence of P_i_ was similar to WT (~13%). When the temperature was increased to 37°C, the proportion of WT cells containing nuclear-localized Crz1-GFP increased to 70% (Fig. 5A and B) consistent with our previous report (30). However, the proportion of *pho80*Δ cells containing nuclear-localized Crz1-GFP increased to only 45% following the temperature shift, indicative of a muted response. By performing a control experiment with DAPI costaining, we confirmed that Crz1 is colocalizing with nuclei in the PHO mutant strains as previously reported (30) for the WT strain (Fig. S1 in the supplemental material).

10.1128/mbio.03551-22.1 Figure S1 Crz1-GFP colocalized with DAPI-stained nuclei in WT, *pho80*Δ, and *pho81*Δ. Cells were grown in YPD at 37°C overnight to promote nuclear localization of Crz1-GFP. As DAPI is not cell permeable, cells were stained with 100 ng/mL DAPI in 0.05% Triton-PBS at room temperature for 5 minutes. Crz1-GFP and DAPI-stained nuclei were visualized using a Delta Vision fluorescence microscope. DIC, differential interference contrast. Download FIG S1, TIF file, 8.1 MB. © Crown copyright 2023. 2023 Crown https://creativecommons.org/licenses/by/4.0/ This content is distributed under the terms of the Creative Commons Attribution 4.0 International license.

P_i_ deprivation increased the proportion of WT and *pho80*Δ cells that contained nuclear localized Crz1-GFP at 25°C (to ~47% for both) (Fig. 5A and B). Shifting the temperature to 37°C caused a further increase in nuclear Crz1-GFP in WT to ~75%. However, the proportion of *pho80*Δ cells containing nuclear Crz1-GFP only increased to 47%, again demonstrating a muted response. The muted calcineurin activation response in *pho80*Δ correlated with the sensitivity of *pho80*Δ to the calcineurin inhibitor, FK506 at 37°C, which was partially rescued by P_i_ depletion (Fig. 5C).

Compared to WT and *pho80*Δ, a higher proportion of *pho81*Δ cells contained nuclear localized Crz1-GFP at 25°C (~57% in the presence of P_i_ and ~84% in the absence of P_i_) (Fig. 5A and B). Irrespective of P_i_ status, the extent of calcineurin activation in *pho81*Δ cells at 37°C was similar to WT at 37°C (Fig. 5A and B). In contrast to *pho80*Δ, *pho81*Δ was more resistant than WT to FK506 at 37°C when P_i_ was present in the growth medium (Fig. 5C). Growth of *pho81*Δ in the presence of FK506 could not be assessed in the absence of P_i_ as this mutant has reduced growth without P_i_ (Fig. 5C). Collectively, our results demonstrate that P_i_ deprivation acts as a general stimulus for calcineurin activation and that increased P_i_ in *pho80*Δ coincides with a muted calcineurin activation response.

### Impact of perturbing P_i_ homeostasis on cellular response to stress.

**FIG 6:**
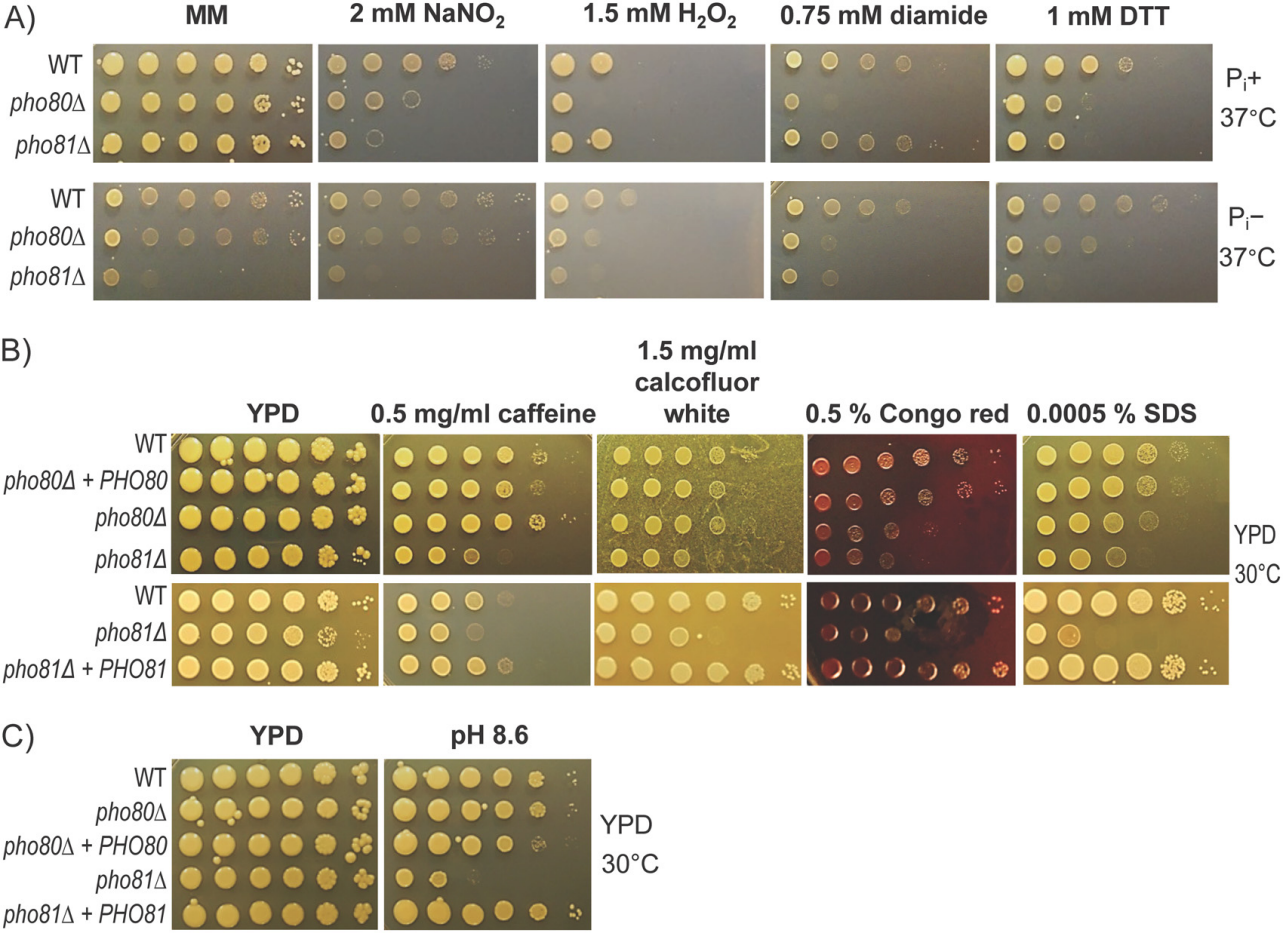
Stress sensitivity is altered by changes in phosphate homeostasis. Strains were grown overnight in YPD and serial 10-fold dilutions were prepared. Three-microliter aliquots of each dilution (containing 10^6^ to 10^1^ cells) were spotted onto MM agar plates containing the indicated concentrations of various stressing agents. (A) Nitrosative, oxidative, thiol oxidative, and thiol reductive stress were tested using 2 mM NaNO_2_, 1.5 mM H_2_O_2_, 0.75 mM diamide, and 1 mM DTT. Growth was assessed for 3 days at 37°C in the presence and absence of P_i_. Control MM plates are duplicated in Fig. 4 and Fig. 5C as drop tests were performed simultaneously. (B) Cell wall stress was tested using 0.5 mg/mL caffeine, 1.5 mg/mL calcofluor white, 0.5% Congo red and 0.0005% SDS. Growth was assessed for 3 days at 30°C in YPD. (C) *pho81*Δ exhibited decreased growth at alkaline pH (pH 8.6) compared to WT and *pho80*Δ.

Metal ion accumulation induces oxidative (H_2_O_2_) and nitrosative (NaNO_2_) stress in fungi (45). We therefore tested the growth of *pho80*Δ, which accumulates a variety of metals, under these stress conditions at the more physiologically relevant incubation temperature of 37°C. Relative to WT, *pho80*Δ growth was hindered by NaNO_2_ stress in P_i_^+^ medium and rescued by P_i_ depletion (Fig. 6A). However, *pho81*Δ, which does not accumulate metals, was more affected than *pho80*Δ to NaNO_2_ stress when P_i_ was present. As P_i_ depletion prevents *pho81*Δ growth, the effect of P_i_ depletion on NaNO_2_ stress could not be assessed. Only *pho80*Δ growth was negatively impacted by H_2_O_2_-induced oxidative stress in the presence of P_i_ and growth was partially rescued by P_i_ deprivation (Fig. 6A). We also tested growth in the presence of diamide and DDT, which are thiol-oxidative and thiol-reductive stress inducers, respectively. *pho80*Δ experienced diamide-induced stress, which was not rescued by P_i_ deprivation. Relative to the WT, both PHO mutants experienced more dithiothreitol (DTT)-induced stress in P_i_^+^ medium and P_i_ deprivation enhanced their growth but also enhanced the growth of WT. In summary, excess or dearth of P_i_ coincides with stress sensitivity. Although stress sensitivity could be due to elevated metal ions in *pho80*Δ, metal ion accumulation is not the cause of this stress sensitivity in *pho81*Δ.

Given that OCR was suppressed in *pho81*Δ (Fig. 2) and mitochondrial function is linked to cell wall integrity (46), we also tested growth in the presence of several cell wall stressing agents. *pho81*Δ was sensitive to caffeine, calcofluor white, Congo red, and SDS (Fig. 6B). However, *pho80*Δ, which has a normal OCR, was also sensitive to Congo red but was mildly resistant to caffeine (Fig. 6B), suggesting that reduced OCR is not responsible for the cell wall defect in *pho81*Δ. Reconstitution of *PHO80* and *PHO81* into *pho80*Δ and *pho81*Δ, respectively, resulted in WT-like phenotypes (Fig. 6B).

Previously, when assessing the virulence of *pho4*Δ in mouse infection models, we demonstrated that a functional PHO pathway is more critical for cryptococcal survival in the blood and hence the central nervous system, than for survival in the lung where C. neoformans grows within acidified cryptococcomas (16, 18). Hence, *pho4*Δ efficiently colonized the lung but not the brain in both inhalation and intravenous mouse models of infection (16). As C. neoformans relies on hydrogen-based P_i_ transporters to acquire P_i_, *pho4*Δ is also sensitive to growth at alkaline pH, which mimics P_i_ deprivation and thus triggers PHO pathway activation even when P_i_ is available (16). Similarly, we now show that *pho81*Δ is sensitive to growth at alkaline pH and that reconstitution of *PHO81* into *pho81*Δ rescued alkaline pH sensitivity (Fig. 6C). In contrast, *pho80*Δ and *pho80*Δ + *PHO80* grow normally at alkaline pH. This is presumably due to the fact that the *pho80*Δ hydrogen-based P_i_ transporters are already upregulated to maintain high intracellular P_i_ reserves.

### Impact of modulating CDK activation on fungal virulence.

**FIG 7:**
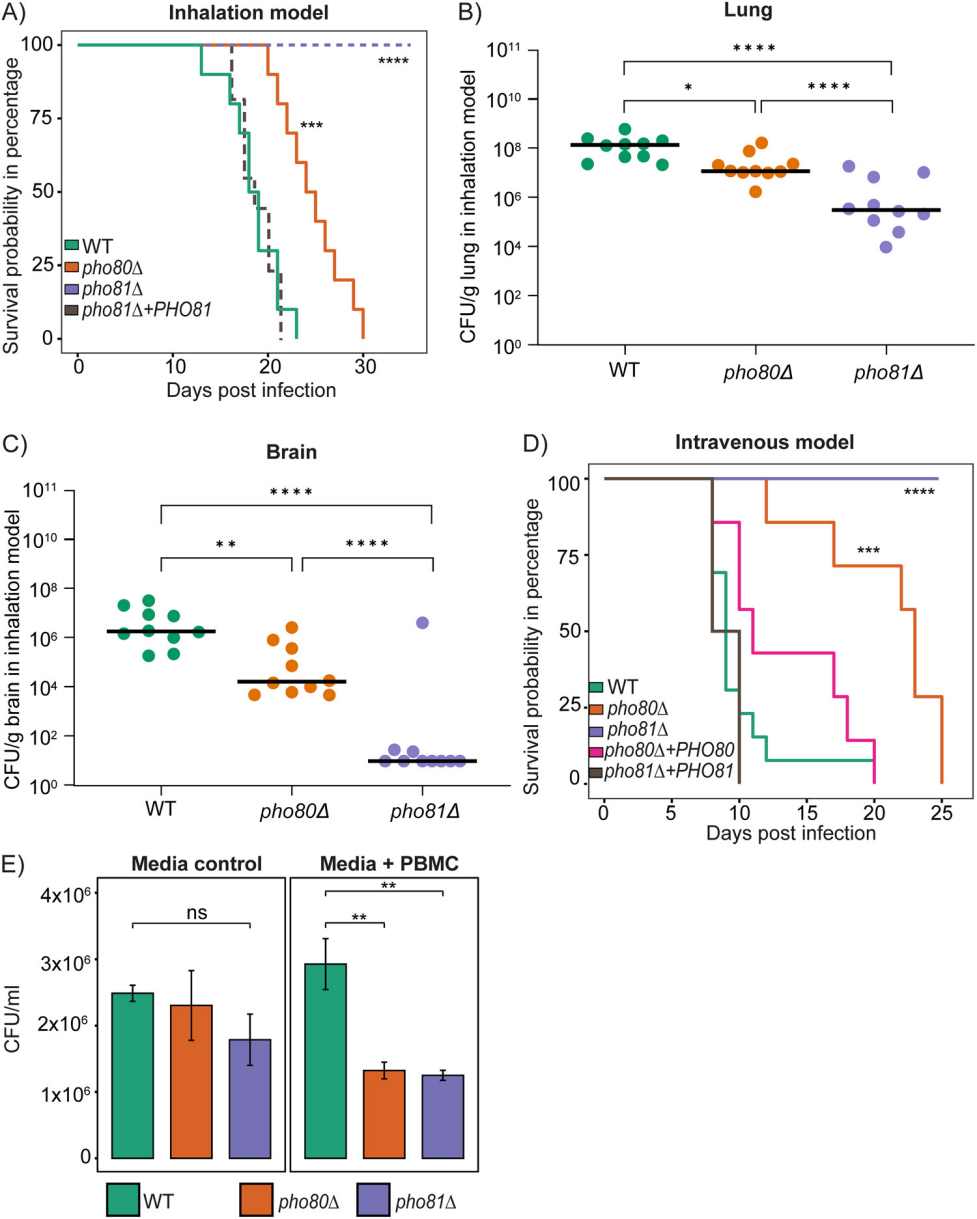
Blocking PHO pathway activation reduces virulence to a greater extent compared to constitutive activation as assessed in mouse and monocyte infection models. (A) *pho80*Δ was less virulent than WT (***, *P* < 0.001) in a mouse inhalation model and the *pho81*Δ strain was avirulent (****, *P* < 0.0001) (*n* = 10 mice per strain). Survival of the *pho81*Δ and *pho81*Δ + *PHO81* strains as shown previously (20) are indicated by a dashed line. Kaplan-Meier survival curves were significantly different and pairwise comparisons were made by the log rank Mantel-Cox test. (B and C) Organ burdens at time of death were lower in *pho80*Δ-infected mice in both the lung (B) and brain (C) compared to the WT-infected mice (*, *P* < 0.05, **; *P* < 0.01; ****, *P* < 0.0001). Organ burdens in *pho81*Δ-infected mice were lower than in WT and *pho80*Δ-infected mice. Colony counts were adjusted to CFU per gram of tissue and graphed as median ± standard deviation. Statistics were determined using a one-way ANOVA and Dunnett’s multiple-comparison test after a log_10_ transformation for normality. (D) *pho80*Δ was less virulent than WT and the *pho80*Δ + *PHO80* strain (***, *P* < 0.001) in a mouse intravenous model, and the *pho81*Δ strain was avirulent (****, *P* < 0.0001) (*n* = 8 mice per strain). Kaplan-Meier survival curves were significantly different and pairwise comparisons were made by the log rank Mantel-Cox test. (E) Coculture of fungal cells with human PBMCs was performed in biological triplicate as described in (16). After incubation with PBMCs for 24 h, survival of *pho80*Δ and *pho81*Δ was reduced compared to WT. Results show the mean ± standard deviation (*n* = 3 biological replicates). Brown-Forsythe ANOVA and Dunnett’s test for multiple comparisons were used to compare difference in growth between mutants under each condition (**, *P* < 0.01; ns, not significant).

C. neoformans infection in mammals is acquired via inhalation to establish a lung infection and can disseminate via the blood to the brain to cause meningoencephalitis. Using a mouse inhalation model to mimic the natural route of infection in humans, *pho80Δ*-infected mice were shown to survive longer than WT-infected mice, with a difference in median survival of approximately 6 days (Fig. 7A). Using the same mouse inhalation model, we previously demonstrated that *pho81*Δ is avirulent, with no *pho81*Δ-infected mice experiencing symptoms of infection or weight loss and all *pho81*Δ + *PHO81*-infected mice succumbing to infection at the same time as the WT-infected mice (median survival ~20 days postinfection) (20). These previously published results for the *pho81*Δ and *pho81*Δ + *PHO81* strains are indicated as dotted lines in Fig. 7A. We also performed organ burden analysis and found a median of 1.5 × 10^8^ CFU per gram of lung for WT-infected mice at the time of death but only 3.7 × 10^5^ CFU per gram of lung for the *pho81*Δ-infected mice at 60 days postinfection (Fig. 7B) (20). However, we found a much higher burden of *pho80*Δ infection at the time of death compared to *pho81*Δ, with 1.4 × 10^7^ CFU per gram of lung (Fig. 7B). A similar trend was observed for brain burdens, with *pho80*Δ-infected mice exhibiting a higher burden of infection at the time of death compared to *pho81*Δ-infected mice at 60 days postinfection (Fig. 7C). In fact, *pho81*Δ was not detected in the brain of 7/10 *pho81*Δ-infected mice at 60 days postinfection.

We also tested the PHO pathway mutants for virulence using an intravenous model. Once again, the *pho80*Δ-infected group survived longer than the WT-infected group and in fact had a longer median survival time than observed in the inhalation model, and *pho81Δ* was once again avirulent (Fig. 7D). Reconstitution of both *pho80Δ* (*pho80Δ + PHO80*) and *pho81Δ* (*pho81Δ + PHO81*) resulted in WT-like virulence (*P* > 0.5).

**FIG 8:**
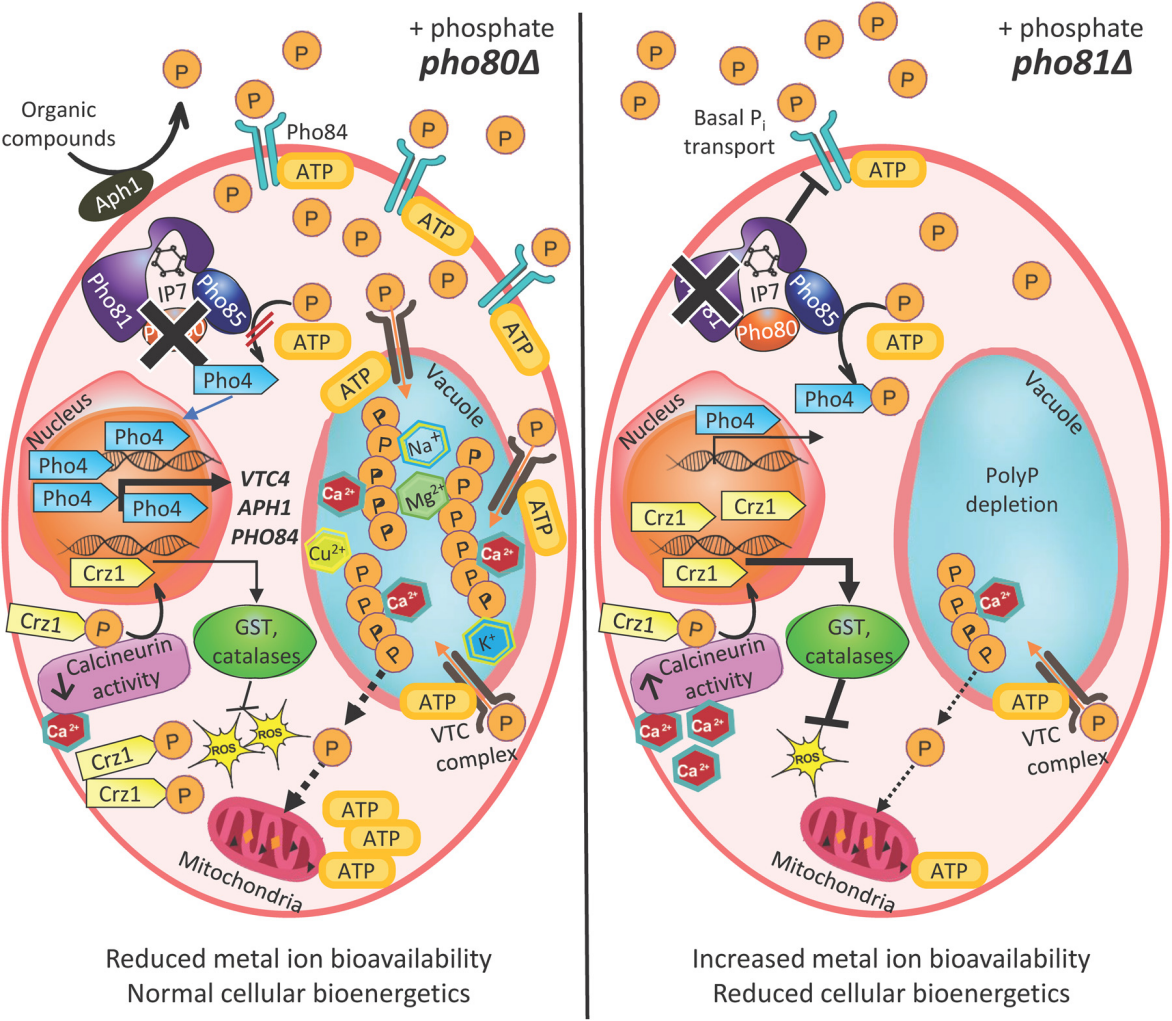
Model of how dysregulated phosphate homeostasis under phosphate-replete conditions impacts calcineurin signaling and energy metabolism in C. neoformans. In *pho80*Δ, the PHO pathway is constitutively activated as Pho85 cannot phosphorylate the transcription factor Pho4 (left). Consequently, the dephosphorylated form of Pho4 dominates and is mostly nuclear localized, leading to enhanced P_i_ mobilization and uptake via Aph1 and Pho84, respectively, and enhanced polymerization of P_i_ into polyP in vacuoles (via the VTC complex). Metal ions accumulate in the vacuole to correct for osmolyte and charge imbalance due to the excess polyP, but this also reduces their bioavailability. Reduced calcium bioavailability in the cytosol leads to a muted calcineurin response and reduced neutralization of reactive oxygen species (ROS). The black dotted line indicates that polyP (from either the vacuole or mitochondria) could be the source of P_i_ for oxidative phosphorylation and ATP generation in mitochondria. In *pho81*Δ, the PHO pathway is repressed, irrespective of P_i_ availability (right). However, intracellular P_i_ is reduced even under P_i_ replete conditions, suggesting that basal import of P_i_ is compromised. Compared to P_i_, ATP and polyP are reduced to an even greater extent. Reduced polyP (from either the vacuole or mitochondria) could negatively impact oxidative phosphorylation and ATP production in mitochondria, contributing further to suppressing ATP-dependent basal P_i_ uptake and polyP polymerization in the vacuole. Increased calcium bioavailability due to reduced sequestration by P_i_ and polyP, increases activity of the phosphatase, calcineurin. This leads to elevated levels of the dephosphorylated (nuclear) form of the transcription factor, Crz1, and enhanced expression of stress response genes, including those encoding glutathione S-transferases (GST) and catalases to combat cellular ROS. The extent of pathway activation is indicated by the degree of boldness of each arrow.

Monocytes play a role in the dissemination of C. neoformans infection from the lung to the brain and are a source of oxidative and nitrosative stress (47–51). We therefore compared the growth of the PHO mutants in coculture with human peripheral blood mononuclear cells (PBMCs). Relative to WT, the growth of both *pho81*Δ and *pho80Δ* were reduced in coculture with PBMCs (Fig. 7E), coinciding with their sensitivity to oxidative and/or nitrosative stress. A model summarizing the phenotypes associated with PHO pathway dysregulation is shown in Fig. 8.

## DISCUSSION

We have shown that Pho80 is the dominant suppressor of PHO pathway activation in C. neoformans, supporting our previous findings (20). Previously, we found that Pho80 and another cyclin, designated Pcl6/7, were associates with Pho85 and Pho81 in immunoprecipitation experiments (20). However, Pcl6/7 was dispensable for PHO pathway activation, instead having a putative role in glycogen metabolism (20). Interestingly, we found that constitutive activation of the PHO pathway in *pho80*Δ in P_i_^−^-replete conditions was not as high as in P_i_^−^-starved WT and that *APH1* can be further activated in *pho80*Δ by P_i_ starvation to achieve levels higher than P_i_^−^-starved WT (Fig. 1A and B). This suggests that a Pho80-independent regulatory mechanism could exist or that loss of Pho80 alters the configuration and/or regulation of the CDK complex via Pho81. In another previous study (16), we found that the CDK inhibitor purvalanol A also uncoupled PHO signaling from P_i_ sensing, leading to constitutive activation of the PHO pathway irrespective of P_i_ availability. Purvalanol A-mediated inhibition of Pho85-Pho80 association should therefore phenocopy *PHO80* deletion. However, given that Purvalanol A is more selective for CDK2 and CDK1 than Pho85 (52, 53), we did not use it in this study, preferring the more targeted approach of *PHO80* deletion to constitutively activate the PHO pathway and allow virulence studies to be undertaken in mice.

Constitutive activation of the PHO pathway in *pho80*Δ led to elevated P_i_ and polyP, which coincided with a mild increase in cellular ATP reserves. Although excess P_i_ could potentially stall metabolism (7), this was not observed, as WT and *pho80*Δ had similar metabolic profiles (Fig. 2B and C). This is consistent with polyP acting as an effective buffer for the excess cytosolic P_i_ in *pho80*Δ. Unexpectedly, the levels of P_i_, ATP, and polyP declined in *pho81* and cellular bioenergetics were suppressed. This was despite P_i_ being present in the growth medium. This contrasts with the WT-like levels of polyP observed in the PHO pathway activation-defective *pho4*Δ when P_i_ was present in the growth medium (16; Fig. S4). Together, these studies suggest that in addition to activating the PHO pathway when basal P_i_ levels drop below ~100 μM (16; Fig. S7), Pho81 is required to maintain basal intracellular P_i_ under P_i_ replete conditions. Hence, the suppressed cellular bioenergetics in *pho81*Δ under P_i_-replete conditions could explain why Pho81 has a more important role in virulence than Pho4. The impact of a dysregulated PHO pathway on P_i_, ATP, and polyP levels and cellular bioenergetics is summarized in Fig. 8.

Understanding how the loss of Pho81 leads to reduced intracellular P_i_ requires further investigation. It could be that the PHO pathway periodically undergoes bursts of activation when P_i_ levels drop temporarily during various phases of the growth cycle, leading to a decrease in cellular P_i_ over time. However, we think that this is unlikely given that we determined the threshold of PHO pathway activation to be as low as 100 μM (16), when cytosolic P_i_ levels are in the order of millimolar. It has been proposed that cytosolic P_i_ levels are conveyed via the binding of 5-PP-IP_5_ to SPX domains (54), and we demonstrated in C. neoformans that 5-PP-IP_5_ binds to the SPX domain of Pho81 to stabilize the CDK complex (20). In S. cerevisiae, basal P_i_ levels are maintained by two low-affinity phosphate transporters (Pho87 and Pho90), each of which has an SPX domain. These transporters are targeted to the vacuole following P_i_ starvation (55, 56). The closest homolog in C. neoformans is CNAG_02180 (Pho91), which encodes a low-affinity vacuolar phosphate transporter. However, the high-affinity transporters Pho84, Pho840, and Pho89 could also contribute to basal P_i_ uptake under P_i_-replete conditions. Although more understanding of the role of Pho81 in basal P_i_ uptake is required, the reduced ATP in *pho81*Δ would slow the import of P_i_ via the basal transport mechanisms as this requires ATP and thus contribute further to the decline in intracellular P_i_.

That polyP and ATP decline to a greater extent in *pho81*Δ compared to P_i_ supports polyP function in C. neoformans extending beyond a role in P_i_ storage to regulating cellular bioenergetics (Fig. 8). In support of our findings, it has recently been reported that S. cerevisiae lacking polyP shows P_i_ starvation features already under P_i_^−^-replete conditions. Based on their metabolomics studies, the authors concluded that vacuolar polyP supplies P_i_ for metabolism even when P_i_ is abundant (57). A reciprocal relationship between ATP and polyP in eukaryotic cells, and a regulatory connection between their relative abundance and synthesis affecting cellular phosphate homeostasis and primary metabolism, have also been reported by others (58). In further support of the connection between polyP and bioenergetics, enzymes responsible for polyP synthesis and utilization have been found in the mitochondria of yeast and mammalian cells (59). Mitochondrial polyP is also a potent regulator of mammalian bioenergetics, with enzymatic depletion of mitochondrial polyP having a deleterious impact on mitochondrial function (60). Although most cellular polyP is stored in the vacuole, up to 10% is located in mitochondrial membranes and the intermembrane space, and increased polyP production in the *pho80*Δ mutant of S. cerevisiae occurred in compartments other than the vacuole (40, 61). Given the evidence in the literature supporting functional and physical communication between mitochondria and lysosome-like organelles in eukaryotes (62), it is also possible that polyP stored in the fungal vacuole regulates mitochondrial function (see model in Fig. 8).

Similar to the *pho80*Δ of S. cerevisiae (40), elevated levels of P_i_ and polyP in the *pho80*Δ mutant of C. neoformans coincided with the accumulation of, and sensitivity to, a broad range of metal ions, and metal ion sensitivity was mostly rescued by P_i_ deprivation (Fig. 4). In contrast, metal ion homeostasis was not affected in *pho81*Δ. The sensitivity of *pho80*Δ to NaCl and KCl, which could also be due to an osmotic stress defect, was also rescued by P_i_ deprivation. Rescue by P_i_ deprivation is consistent with these phenotypes being conveyed via Pho4.

High levels of P_i_ and polyP in *pho80*Δ, leading to the formation of metal-phosphate complexes, could negatively impact cellular function by limiting metal bioavailability. In support of this, serum calcium levels are raised in humans during hyperphosphatemia leading to calcification in the arteries (63, 64). The *pho80*Δ mutant is therefore essentially a cellular model of hyperphosphatemia, where the increase in cellular calcium is not necessarily bioavailable due to reduced solubility. Furthermore, our data support elevated P_i_/polyP as the cause of the observed metal ion accumulation and the metal hypersensitivity in *pho80*Δ since these phenotypes are rescued by the removal of phosphate. Reduced metal ion bioavailability could subsequently lead to the generation of reactive oxygen species (ROS) (39). For example, the sequestration of metal ions that act as cofactors for copper/zinc superoxide dismutase Sod1 (26), would generate oxidative stress, and calcium sequestration could suppress calcineurin activation as observed in *pho80*Δ, thereby reducing the expression of genes involved in mitigating oxidative stress (30–32). The oxidative stress sensitivity in *pho80*Δ created by a combination of a muted calcineurin response and metal accumulation (summarized in Fig. 8) is likely to negatively impact its survival in the presence of monocytes and its virulence in mouse models (Fig. 7). Elevated metal ions in *pho80*Δ also coincided with other chemical stress sensitivities. However, *pho81*Δ shared some of these stress sensitivities, including to nitrosative and DTT stress but not oxidative stress or diamide stress. These stress sensitivities in *pho81*Δ could be due to reduced mitochondrial function.

Attenuated virulence of *pho80*Δ could also potentially be due to blocked phosphorylation of Pho80-Pho85 substrates other than Pho4. Other Pho80-Pho85 substrates in S. cerevisiae include Crz1 and Rim1 (42, 43). However, given the modest attenuation in *pho80*Δ virulence, particularly in the inhalation model, our results suggest that Pho80-Pho85 substrate specificity may be more restricted in C. neoformans, with Pho4 (and hence the PHO pathway) being the major target of Pho80-Pho85. Furthermore, the expansion of PHO gene targets in C. neoformans to include transporters of nutrients other than P_i_ could potentially promote adaptation to a host environment (18).

We also further explored a regulatory connection between P_i_ availability and calcineurin activation using a set of calcineurin reporter strains in a WT (30) and *pho80*Δ and *pho81*Δ background. The results show that (i) the calcineurin activation response was muted in P_i_/polyP-enriched *pho80*Δ as indicated by less nuclear Crz1 and alleviation of FK506 sensitivity by P_i_ depletion, (ii) the calcineurin pathway was hyperactivated in the P_i_/polyP-deficient *pho81*Δ mutant in the absence of heat stress (25°C) and further activated at 25°C by P_i_ depletion, and (iii) P_i_ deprivation stimulated calcineurin activation in WT in the absence of heat stress (more Crz1 is nuclear). P_i_ deprivation is therefore a general stimulus for calcineurin activation and excess P_i_ suppresses calcineurin activation. Given that elevated extracellular calcium can trigger Crz1 to migrate to the nucleus (30), we considered the possibility that the muted calcineurin response in *pho80*Δ is due to reduced calcium bioavailability caused by P_i_ and polyP-mediated calcium sequestration. However, the interpretation of the findings in this mutant is complicated, given that Crz1 could also be a substrate of Pho85-Pho80. In S. cerevisiae, Crz1 is a substrate of Pho85-Pho80, and phosphorylation of Crz1 by Pho85-Pho80 acts as a negative regulator of Crz1 nuclear localization and, hence, calcineurin activation (42, 43). If the same is true for C. neoformans, Crz1 would not be phosphorylated by Pho85 in *pho80*Δ and a higher ratio of nuclear to cytoplasmic Crz1 would be expected, when in fact we found the opposite. We therefore considered the opposite scenario in that increasing calcium bioavailability through P_i_ deprivation, which is achieved by growing WT without P_i_ or deleting *PHO81* (*pho81*Δ), would enhance calcineurin activation, and we found this to be the case (Fig. 5). Thus, based on a range of experiments using three different strains (*pho80*Δ, *pho81*Δ, and WT), only one of which has a metal ion homeostasis defect, our data support P_i_ availability impacting the calcineurin activation response by altering calcium bioavailability (see model in Fig. 8). This is consistent with P_i_ availability impacting calcium bioavailability in patients with renal dysfunction and hyperphosphatemia (63, 64). It is also possible that excess P_i_ and ATP in *pho80*Δ favors phosphatase activity, including calcineurin, over kinase activity. This would result in a higher ratio of phosphorylated to dephosphorylated Crz1 in *pho80*Δ and, hence, a higher proportion of cytosolic Crz1 as we observed (Fig. 5). In contrast, the lower P_i_ and ATP in *pho81*Δ would favor calcineurin activation due to a higher proportion of dephosphorylated, and hence nuclear, Crz1 as we observed (Fig. 5). The influence of P_i_ levels on calcineurin activation has, to the best of our knowledge, never been demonstrated.

In summary, blocking, as opposed to permanently activating, the PHO pathway reduces fungal growth in host tissues and virulence to a greater extent. The differential response is most likely attributable to *pho81*Δ having depleted P_i_ and polyP and compromised cellular energetics, irrespective of P_i_ availability, in addition to the stress sensitivities it shares with *pho80*Δ. Using several approaches, we also show that P_i_ deprivation most likely stimulates calcineurin activation by enhancing metal ion availability. Our studies are useful in informing drug-targeting strategies centered around this fungal-specific process and suggest that targeting the CDK inhibitor Pho81, which has no homolog in humans, to prevent association with Pho85-Pho80, is a preferable antifungal strategy to targeting Pho85-Pho80 association.

## MATERIALS AND METHODS

### Growth conditions.

Standard growth conditions used were YPD (1% yeast extract, 2% peptone, and 2% glucose) at 30°C with shaking at 250 rpm in a volume that was ≤20% of the maximum container capacity to allow for aeration. Some experiments used minimal medium (MM: 15 mM glucose, 10 mM MgSO_4_·7H_2_O, 13 mM glycine, and 3 μM thiamine) either supplemented with 29 mM KH_2_PO_4_ (MM-KH_2_PO_4_ or P_i_^+^ medium) or supplemented with 29 mM KCl (MM-KCl or P_i_^−^ medium). Some experiments used low phosphate YPD (LP-YPD) in which phosphate is depleted by precipitation with concentrated NH_4_OH as described previously (20).

### Strain construction.

The strains used in this study are listed in Table 1. Primers used to create constructs for homologous recombination and to verify targeted gene deletion and tagging are listed in Table 2. The biolistic transformation of C. neoformans was originally described in reference 65 and performed as described in reference 66.

**TABLE 1:** Strains used in this study^*a*^

Strain name	Background	Genotype	Gene identification	Reference
WT	Cryptococcus neoformans *var. grubii* strain H99		NA	
*pho80*Δ	H99	*pho80Δ::NEO*	CNAG_01922	This study
Crz1-GFP (calcineurin) reporter strain (Nat^r^)	H99	*Crz1-GFP-NAT*	CNAG_00156	This study
*pho80*Δ Crz1-GFP (calcineurin) reporter strain	WT Crz1-GFP-NAT	*pho80*Δ::*NEO* *Crz1-GFP-NAT*	CNAG_01922 CNAG_00156	This study
*pho80*Δ + *PHO80*	*pho80*Δ	*pho80Δ::NEO* *PHO80-NAT*	CNAG_01922	This study
*pho81*Δ	H99	*pho81Δ::HYG*	CNAG_02541	20
*pho81*Δ + *PHO81*	*pho81*Δ	*pho81Δ::HYG* *PHO81-NEO*	CNAG_02541	20
Crz1-GFP (calcineurin) reporter strain (Neo^r^)	H99	*Crz1-GFP-NEO*	CNAG_00156	30
*pho81*Δ Crz1-GFP (calcineurin) reporter strain	WT Crz1-GFP-NEO	*pho81Δ::NAT* *Crz1-GFP-NEO*	CNAG_02541 CNAG_00156	This study

a Tabulated strain name, background strain, and selection marker used for strain construction are shown.

**TABLE 2:** Primers used for strain construction and validation^*a*^

Primer name	Sequence (5′ to 3′)	Primer name	Sequence (5′ to 3′)	Description
PHO80 ots s	AATCCTGCGCCTGGTCAACAA	PHO80 5′a	*CTCCAGCTCACATCCTCGCAG*GTATTGGCAAGGCATTATTA	*PHO80* deletion; 5′flank of *PHO80*
PHO80 3′s (NEO)	*CCTCAGGATCTTCATGGCTCC*ATGCATACAACTTCAACACC	PHO80 ots a	TCGTCTTTGACACTGCGCCC	*PHO80* deletion; 3′flank of *PHO80*
PHO80 5′s	ATTGAACGACGACCGACAGCAT	PHO80 3′a	TCCCATGATCCAGCAGGTGAGT	*PHO80* deletion; overlap PCR to create *PHO80:NEO*
Neo-s	CTGCGAGGATGTGAGCTGGAG	Neo-a	GGAGCCATGAAGATCCTGAGG	Amplification of neomycin and nourseothricin resistance cassettes
PHO80-s	ATAACCTGCGTCAACCTCCCGA	PHO80-a	ATGTCGGTGTCGGCTGGTTCA	PHO80 expression
PHO80 ots s	AATCCTGCGCCTGGTCAACAA	ActP-a	TGTTGTTACCATCATCCTCTCCTC	Verification PCR to confirm *PHO80* deletion, external 5′ recombination
Ttrp-s	CTACAGACAACAATACCATCCTTCC	PHO80 ots a	TCGTCTTTGACACTGCGCCC	Verification PCR to confirm *PHO80* deletion, external 3′ recombination
Neo-s	CTGCGAGGATGTGAGCTGGAG	HygB a	TCTCTATACGGCGATTGGCGGA	Hygromycin resistance cassette
PHO80 Rec s	AATAGAGTCCAAGAAGAGCGTGCCC	PHO80(NAT) Rec a2	*CTCCAGCTCACATCCTCGCAG*CAGTTGGTGATAGCGGGTGG	*PHO80* gene amplification for reconstitution with nourseothricin resistance gene (NAT^r^) as marker
PHO80 Rec s2	TCACCTCTCCAATACTAATCACCGA	Neo-a	GGAGCCATGAAGATCCTGAGG	Overlap PCR to fuse PHO80 gene with NAT^r^ gene.
PHO81 ots s	ATGTATTATCCTGCTCTGTCGCTCCA	PHO81 5′ a	*CTCCAGCTCACATCCTCGCAG*CAACTGGCTGGAGATAAAGC	*PHO81* deletion; 5′flank of *PHO81*
Pho81-3′ NAT s	CCTCAGGATCTTCATGGCTCCCATGTAACTGTAATACTAGC	PHO81 ots a	GTATCAACTCAAACTCTTCGGCAGGA	*PHO81* deletion; 3′flank of *PHO81*
PHO81 5′s	CCCATACTTGCCTTCATACCCTTCAG	PHO81 3′a	GCGATTGATTGATGAGGGATAGGG	Overlap PCR to create *PHO81:NAT* construct
PHO81 ots s	ATGTATTATCCTGCTCTGTCGCTCCA	ActP-a	TGTTGTTACCATCATCCTCTCCTC	Verification PCR to confirm *PHO81* deletion, external 5′ recombination
Ttrp-s	CTACAGACAACAATACCATCCTTCC	PHO81 ots a	GTATCAACTCAAACTCTTCGGCAGGA	Verification PCR to confirm *PHO81* deletion, external 3′ recombination

a Italics in the sequences indicate section that corresponds to the resistance marker primer.

**(i) *PHO80* deletion and reconstitution.** The deletion construct for *pho80*Δ consisted of a 5'-flank, neomycin resistance (Neo^r^) cassette and a 3' flank, and 1,238 bp of genomic DNA upstream of the *PHO80* coding sequence was amplified using primer pair PHO80 ots s with PHO80 5'a. The Neo^r^ cassette (with the *ACT1* promoter and *TRP1* terminator) was amplified from pJAF (67) using primer pair Neo-s and Neo-a, and 1,095 bp of genomic DNA downstream of the *PHO80* coding sequence was amplified using primer pair PHO80 3′s (NEO) with PHO80 ots a. The three fragments were joined by overlap PCR using primer pair PHO80 5′s with PHO80 3′a and then used to transform H99 WT using biolistics (20). Transformants that were resistant to neomycin were screened by PCR across the 5' and 3' junctions using primer pair PHO80 ots s with ActP-a and primer pair Ttrp-s with PHO80 ots a. Positive colonies were screened for acid phosphatase (Aph1) activity using the colorimetric pNPP reporter assay as previously described (16). The *PHO80* reconstituted strain (*pho80*Δ + *PHO80*) was created by PCR amplifying the 809-bp coding region (CNAG_01922) with 883 bp upstream and 678 bp downstream, from H99 WT genomic DNA using primer pair PHO80 Rec s and PHO80(NAT) Rec a2. The nourseothricin resistance (Nat^r^) cassette (with the *ACT1* promoter and *TRP1* terminator) was PCR amplified using the primer pair Neo-s and Neo-a. The two fragments were fused by overlap PCR using primer pair PHO80 Rec s2 and Neo-a and then used to transform *pho80*Δ. Colonies resistant to nourseothricin were screened for acid phosphatase (Aph1) activity as above. Transformants that reverted to the WT phenotype, i.e., minimal acid phosphatase activity in the presence of P_i_ were further tested for incorporation of the *PHO80* gene by PCR amplification of an internal region of the *PHO80* locus using primer pair PHO80-s and PHO80-a. Expression of *PHO80* was also compared to WT and *pho80*Δ using qRT-PCR with primers PHO80-s and PHO80-a and was restored to 80% of the WT expression level.

**(ii) GFP-tagging Crz1.** *(a) In PHO81 deletion background*. A *PHO81* deletion construct (*pho81*Δ*:NAT*) was created, which consisted of a 5'-flank, a nourseothricin resistance (Nat^r^) cassette and a 3' flank. To make this deletion construct, 963 bp of genomic DNA upstream of the *PHO81* coding sequence was PCR amplified using primer pair PHO81 ots s and PHO81 5′a. The nourseothricin resistance (Nat^r^) cassette (with the *ACT1* promoter and *TRP1* terminator) was PCR amplified using the primer pair Neo-s and Neo-a, and 1,402 bp of genomic DNA downstream of the *PHO81* coding sequence was PCR amplified using primer pair Pho81-3′ NAT s and PHO81 ots a. The three fragments were joined by overlap PCR using primer pair PHO81 5′s and PHO81 3′a. This overlapping fragment was then used to transform the *Crz1-GFP-NEO* WT strain (30) using biolistic transformation. Nourseothricin-resistant *PHO81* deletion colonies were confirmed by PCR amplification across the 5′ and 3′ junctions using primer pair PHO81 ots s and ActP-a, and primer pair Ttrp-s and PHO81 ots a. Transformants were screened for acid phosphatase (Aph1) activity to confirm that they had the same phenotype as *pho81*Δ, i.e., no elevated Aph1 activity in the absence of P_i_.

*(b) In PHO80 deletion background.* The Nat^r^ cassette was used to transform the *Crz1-GFP-NEO* WT strain (30) to replace NEO with NAT, creating a *Crz1-GFP-NAT* WT strain (Table 1). The *PHO80* deletion construct (created as described above) was then used to transform the *Crz1-GFP-NAT* WT strain using biolistic transformation. Insertion of the *PHO80* deletion construct in neomycin-resistant colonies was confirmed by PCR amplification across the 5' and 3' junctions as above. Transformants were screened for acid phosphatase (Aph1) activity as above to confirm that they had the same phenotype as *pho80*Δ, i.e., elevated Aph1 activity in the presence of P_i_.

**Assessing the PHO pathway activation.** Overnight cultures were grown in YPD media, at 30°C with shaking (250 rpm). Cells were pelleted by centrifugation, washed twice with water, and adjusted to an optical density at 600 nm (OD_600_) = 1 by resuspending the pellet in either MM-KH_2_PO_4_ (P_i_^+^) or MM-KCl (P_i_^−^). The cultures were incubated at 30°C with shaking (250 rpm) for 3 h. The cultures were used for assessing PHO pathway activation by qRT-PCR and acid phosphatase assay.

**(i) Acid phosphatase assay.** This assay measures the extracellular acid phosphatase activity attributable to secreted Aph1 (product of the *APH1* gene). Aliquots of 20 μL were added to a reaction mixture of 50 mM sodium acetate (pH 5.2) and 2.5 mM p-nitrophenyl phosphate (p-NPP), a colorimetric substrate of Aph1, in a final volume of 400 μL. Samples were incubated at 37°C for 10 min, and the reaction was stopped by addition of 800 μL of 1 M Na_2_CO_3_. Cells were pelleted by centrifugation and the absorbance of the supernatant was measured at 420 nm, which is the absorbance maxima of the pNPP hydrolysis product. Cultures were also grown simultaneously to assess growth by measuring optical density at 600 nm. The activity was then corrected for growth differences by expressing it as a ratio (A_420_/A_600_). Samples were prepared in biological triplicate and analyzed by two-way ANOVA with Tukey’s test for multiple comparisons.

**(ii) qRT-PCR.** Cultures were incubated as above and then pelleted by centrifugation, and total RNA was extracted using TRIzol Reagent (Invitrogen) as per the manufacturer’s instructions. cDNA was prepared using Moloney murine leukemia virus reverse transcriptase (Promega) with Oligo(dT) primers. Gene expression was quantified by qRT-PCR using Platinum SYBR green qPCR SuperMix-UDG (Life Technologies, Inc.) in a CFX-96 Touch Real-Time PCR Detection System (Bio-Rad Laboratories). The primers used are listed in Table 3. Fold change in target gene expression was normalized to actin (*ACT1*) as a reference gene. Data were analyzed with CFX Maestro, and the FC = 2^−ΔΔCt^ method was used to calculate fold change in expression between samples relative to WT incubated in P_i_ conditions at 25°C.

**TABLE 3:** Primers used for assessing PHO gene expression and gene expression impacted by TOR and calcineurin pathway inhibition using qRT-PCR^*a*^

Target gene	Sense sequence	Antisense sequence
*ACT1* actin (CNAG_00483)	ATGGTATTGCCGACCGTATG	CTCTTCGCGATCCACATCTG
*PHO89* sodium-dependent phosphate transporter (CNAG_05075)	GTGCTCGGTAACAGACTGAC	ACGCTCGCCAGTTAATCG
*VTC4* vacuolar chaperone complex 4 (CNAG_01263)	GATGCCGTCGGTATGGTTTC	TAACAACGCCGCGCAAAG
*BTA1* betaine lipid synthase (CNAG_02353)	CCCATTCCAACGCTTTCTACTCTCA	AGCGACTCATCAGGAAGACCCC
*PHO84* phosphate:H symporter (CNAG_02777)	CCTACTCGTTACCGATCAACTG	AGTCTCGGGAAGAAGCAATG
*APH1* phosphate-repressible vacuolar acid phosphatase (CNAG_02944)	CCTTCAACTTGAGTGCGCTT	ACATCCCGTCCTTGTTCCAT
*PHO81* cyclin-dependent protein kinase inhibitor (CNAG_02541)	AAGAAGGTAGGAAGGGAGAGCGG	ATGAAGATTGACGGGAGACGCC

a TOR, target of rapamycin.

**Analysis of cellular orthophosphate and polyphosphate.** Overnight cultures of WT H99, *pho80Δ*, and *pho81Δ* cells were grown in YPD media, at 30°C with shaking (250 rpm). Cells were pelleted by centrifugation, washed twice with water, and adjusted to OD_600_ = 1 in 30 mL of either MM-KH_2_PO_4_ (P_i_^+^) or MM-KCl (P_i_^−^) medium and incubated for 3 h at 30°C. Cells were pelleted and snap frozen in liquid nitrogen. Cells were thawed in 600 μL of LETS buffer (100 mM LiCl, 10 mM EDTA, 10 mM Tris-HCl, pH 8, and 0.5% SDS) per sample of OD_600_ at 30. An equal volume of acid phenol was combined and approximately 400 μL of 450 to 600 μm acid-washed glass beads was added. Cells were homogenized at 4°C with the MiniBeadbeater-8 cell disrupter (Daintree Scientific, Tasmania, Australia): 4 × 30-sec cycles with 1-min intermittent resting at 4°C between cycles. The lysates were centrifuged at 14,000 × *g* at 4°C. Approximately 20% of the supernatant was reserved for free P_i_ quantification and was stored at −80°C. PolyP was precipitated from the remaining supernatant by adding 2.5 volumes of absolute ethanol, mixing by inversion, and incubating at −80°C overnight.

**(i) Free phosphate quantification.** Samples were thawed, and free P_i_ was quantified using the Phosphate Colorimetric kit (Sigma-Aldrich) following the manufacturer’s instructions. Samples were prepared in biological duplicate, and results were analyzed by one-way ANOVA with Dunnet’s test for multiple comparisons against WT H99.

**(ii) PolyP quantification.** PolyP was extracted and quantified using the method described in (68). Briefly, ethanol-precipitated polyP was pelleted by centrifugation at 14,000 × *g* at 4°C, resuspended in UltraPure water, and normalized to RNA concentration. Samples and a polyP standard were loaded onto a 3% MetaPhor agarose gel and run at 70 V for 90 min. Gels were stained with 0.01% wt/vol toluidine blue (in 20% methanol, 2% glycerol) for 30 min to visualize the polyP and RNA and destained overnight in water.

**ATP quantification.** Cells were grown in YPD overnight, then washed twice with water, and resuspended in 10 mL of MM-KH_2_PO_4_ (P_i_^+^) at OD_600_ = 1. Cells were further incubated at 30°C, 250 rpm for 3 h and then pelleted by centrifugation. Cell pellets were snap frozen in liquid nitrogen and stored at −30°C. Cell lysates were extracted by resuspension in 500 μL 50 mM HEPES, pH 7.7 and then bead beaten for five cycles of 45 sec with at least 1 min of cooling on ice between samples. Samples were centrifuged at 2,500 × *g* for 10 min at 4°C. The supernatant was collected for ATP quantification using the Sigma ATP Bioluminescent assay kit and for protein estimation using the BCA Protein assay kit.

**Measuring metabolism using the Seahorse analyzer.** This method was carried out as previously described (36). Briefly, YPD overnight grown cultures were washed twice with water and resuspended in assay medium (Seahorse XF Base medium supplemented with 2 mM l-glutamine and 5 mM HEPES, pH 7.4). A 500 μL volume of cells (OD_600_ = 0.04) was seeded per poly-l-lysine coated well and centrifuged twice in different orientations at 300 × *g* for 5 min with minimal acceleration and deceleration. Each well was photographed under ×4 magnification to obtain a measurement of confluence to normalize the data between wells. Using the Seahorse XFe24 Analyzer instrument (Agilent), the basal metabolic activity was measured for up to 40 min at 37°C. Changes in metabolic activity were measured for 80 min following the addition of each compound: 20 mM glucose (final concentration), 50 μM antimycin A, 50 μM rotenone, and 100 mM 2-deoxy-d-glucose.

**Metal analysis using inductively coupled plasma mass spectrometry.** Strains were grown overnight in YPD or LP-YPD at 37°C with 250 rpm and a starting OD_600_ of 0.1 per mL. Cells were pelleted and washed twice in 50 mM Tris-HCl, pH 7.5 and then incubated in 10 mM EDTA and 50 mM Tris-HCl, pH 7.5 for 5 min at room temperature to remove surface-bound metals. Cells were pelleted and washed twice in 50 mM Tris-HCl, pH 7.5 to remove EDTA and then incubated for 24 h in 1 mL 65% nitric acid at room temperature. Cells were centrifuged at 13,000 rpm for 5 min to remove any debris, and the supernatant was diluted 10-fold in UltraPure water and analyzed by ICP-MS with a Cetac ASK 520 autosampler. Biological triplicates were analyzed for sodium (^23^Na), magnesium (^24^Mg), aluminum (^27^Al), phosphorus (^31^P), potassium (^39^K), calcium (^43^Ca), titanium (^47^Ti), chromium (^52^Cr), manganese (^55^Mn), nickel (^60^Ni), copper (^63^Cu), and zinc (^66^Zn). Samples were analyzed on a PerkinElmer Nexion 300X ICP-MS in kinetic energy discrimination mode (4 mL/min He collision gas). Isotopes were analyzed with a 1-s integration time for all elements measured. A 10-ppb Rh/Ir internal standard solution was added to the flow system to correct for plasma energy variation. Argon plasma flow rate was 16 L/min with an ICP power of 1.5 kW. Concentrations were calculated by comparison to elemental standards analyzed within the same run and normalized to OD_600_ for each culture. Statistically significant differences were identified by one-way ANOVA with Sidak’s test for multiple comparisons on GraphPad Prism 9. For each element, each strain was compared against each other in YPD, each strain was compared against each other in LP-YPD, and each strain was compared against itself in YPD versus LP-YPD.

**Growth tests for susceptibility to various stressors.** Strains were cultured overnight in YPD. Cell concentration was adjusted to 10^6^ cells per spot volume (usually 3 μL) and 10-fold serial dilutions were prepared over a range of 10^6^ to 10^1^ cells per spot volume. Cell dilutions were spotted onto various media with different chemicals, including 500 μM CuSO_4_, 0.5 M NaCl, 1.2 mM NiCl_2_, 0.6 M KCl, 100 mM CaCl_2_, 1 μg/mL FK506, 2 mM NaNO_2_, 1.5 mM H_2_O_2_, 0.75 mM diamide, 1 mM DTT, 0.5 mg/mL caffeine, 1.5 mg/mL calcofluor white, 0.5% Congo red, and 0.0005% SDS at pH 8.6. Plates were incubated at 25°C, 30°C, or 37°C as indicated and photographed at 72 h to record macroscopic growth.

**Fluorescence microscopy to visualize Crz1.** Strains were grown overnight in YPD media. Cells were pelleted by centrifugation, diluted 1/5 in MM-KH_2_PO_4_ (P_i_^+^) or MM-KCl (P_i_^−^) medium and then incubated at 25°C or 37°C for 4 h. Images were collected using a UPLSAPO ×100 oil (1.4NA) objective on a Deltavision Elite microscope (Applied Precision, GE Health) attached to a CoolSNAP HQ2 CCD camera with exposure times of 0.5 sec for polarised light and 1.0 sec for FITC. Deconvolution and image processing was performed using SoftWoRx Suite 2.0 (Applied Precision, GE Health) and ImageJ (69). During postprocessing, the same scale was used for all conditions.

**Virulence studies in mouse models.** Female mice (C57BL/6) were sourced from the Australian Resource Centre (Western Australia) at 6 to 8 weeks old. Access to food (autoclavable rat and mouse chow by Specialty Feeds) and water was unrestricted. The light-dark cycle was 12 h. Mice were acclimatized for 1 week before experiments commenced. All mice experiments were undertaken according to protocol 4254.03.16 as approved by the Western Sydney Local Health District animal ethics committee.

**(i) Mouse inhalation model.** Ten mice per strain were anesthetized by inhalation of 3% isoflurane in oxygen and infected with 2 × 10^5^ cells via the nasal passages as described previously (20). Mice were monitored daily and euthanized when they had lost 20% of their preinfection body weight or if showing debilitating symptoms, such as labored breathing or severe imbalance. Differences in median survival between infection groups were analyzed by Kaplan-Meier log-rank (Mantel-Cox) test using SPSS (Version 26) statistical software. Lungs and brain were collected and mechanically disrupted in 1 mL PBS using 2-mm zirconium beads and the BeadBug homogenizer (Benchmark Scientific). Homogenized organs were serially diluted and plated onto Sabouraud dextrose agar (SAB) plates and incubated at 30°C for 2 days. Colony counts were adjusted according to organ weight and differences in fungal burdens were determined by one-way ANOVA and Dunnett’s multiple-comparison test of log_10_ transformed data using GraphPad Prism 9.

**(ii) Mouse dissemination model.** Eight mice per strain were anesthetized by inhalation of 3% isoflurane in oxygen and infected intravenously with 5 × 10^3^ cells via the retro-orbital plexus as described previously (16). Mice were monitored and euthanized, and survival was analyzed as above.

**Monocyte/fungal coculture experiments.** Fungal cells were grown overnight in YPD, washed twice in water, and labeled with 0.5 mg/mL fluorescein isothiocyanate (FITC) (Sigma) in phosphate-buffered saline (PBS) for 30 min with shaking. Excess stain was removed by washing the fungal cells several times in PBS and then resuspending the final pellet in RPMI-FBS medium. Fungal cells (5 × 10^5^) were opsonized for 30 min in 25 μL 75% human serum and 200 ng of the opsonizing antibody 18B7, which targets glucuronoxylamannan (GXM; a gift from Arturo Casadevall, Johns Hopkins, MD). PBMCs were isolated from human blood using Ficoll-Paque Plus (GE Healthcare) following the manufacturer’s instructions and resuspended in RPMI-FBS. Fungal cells (5 × 10^5^) from each strain were incubated alone as a control or coincubated with 5 × 10^5^ PBMCs in 200 μL RPMI-FBS at 37°C with 5% CO_2_ for 24 h. PBMCs were lysed by the addition of 0.05% SDS, and fungal cells were serially diluted and plated on SAB plates. CFU were counted after 2 days of growth at 30°C.
